# Akt-Fas to Quell Aberrant T Cell Differentiation and Apoptosis in Covid-19

**DOI:** 10.3389/fimmu.2020.600405

**Published:** 2020-12-21

**Authors:** Anthony J. Leonardi, Rui B. Proenca

**Affiliations:** ^1^Johns Hopkins Bloomberg School of Public Health, Baltimore, MD, United States; ^2^Department of Biology, Johns Hopkins University, Baltimore, MD, United States

**Keywords:** Akt, Fas, T-cells, apoptosis, lymphopenia, exhaustion, COVID-19, differentiation

## Abstract

Aberrant T cell differentiation and lymphopenia are hallmarks of severe COVID-19 disease. Since T cells must race to cull infected cells, they are quick to differentiate and achieve cytotoxic function. With this responsiveness, comes hastened apoptosis, due to a coupled mechanism of death and differentiation in both CD4+ and CD8+ lymphocytes *via* CD95 (Fas) and serine-threonine kinase (Akt). T cell lymphopenia in severe cases may represent cell death or peripheral migration. These facets depict SARS-Cov-2 as a lympho-manipulative pathogen; it distorts T cell function, numbers, and death, and creates a dysfunctional immune response. Whether preservation of T cells, prevention of their aberrant differentiation, and expansion of their population may alter disease course is unknown. Its investigation requires experimental interrogation of the linked differentiation and death pathway by agents known to uncouple T cell proliferation and differentiation in both CD4+ and CD8+ T cells.

## Introduction

A remarkable feature of infection identified early in the pandemic was a relative lymphopenia in patients shown to correlate with poor outcomes (1–4). Whether this was due to direct viral infection, activation-induced cell death or migration to the periphery remains unknown. Severe Covid-19 is associated with significant lymphocyte hyperactivation, organ infiltration, and tissue damage, suggesting widespread and, perhaps, deleterious immune activity. T-cell differentiation and collapse can be facilitated by the canonical death receptor CD95 and interestingly, Bellesi et al. describe the increased expression of CD95 and PD-1 on the T lymphocytes of Covid-19 patients and suggest it could predispose T cells to apoptosis and exhaustion (5). Here, we explore the implications of targeting lymphocyte death and differentiation in Covid-19 *via* Fas/Akt, and we propose that CD95 may not only serve as a functional marker for apoptosis but also a means of exuberant differentiation culminating in cytotoxic function and this expression may represent a therapeutic point of intervention.

## Discussion

### Alternative Hypotheses to the “Cytokine Storm” Needed

The immune dysfunction in Covid-19 has been so far enigmatic. At first, the focus was on the elevation of cytokines characteristic of the innate immune compartment (a “cytokine storm”) associated with severe Covid-19, including IL-6, IL-8, and IL-10, among others (3, 4, 6–8). However, a meta-analysis showed IL-8 and IL-10 were substantially lower in Covid-19 as compared to conditions like cytokine release syndrome, Acute Respiratory Distress Syndrome (ARDS), and Sepsis (7). The authors suggest the attribution of a “cytokine storm” to Covid-19 pathogenesis was questionable (7). In a randomized trial, IL-6 blocking therapy was unfortunately noted to be of little therapeutic benefit in hospitalized patients (9). Based on their findings, Leisman, et al. call for alternative hypothetical mechanisms explaining the level of organ damage and immunopathology (7). One consistency they note, however, is the preponderance of lymphopenia in cases of severe Covid-19 (7).

#### Dissecting Possible T Cell Migration, “Exhaustion”, and End Organ Damage in Covid-19

A key question is whether the lymphopenia is a mere marker of worsening disease or is a mechanistic component where intervention could influence outcomes. Diao et al. and Wang et al. note decreases in CD4+ and CD8+ T cells (10, 11), and Wang et al. show in a multivariate analysis that posttreatment decreases in CD8+ T cells, B cells, and an increased CD4+/CD8+ ratio were independent predictors of poor outcomes (11). Another study by Moderbacher et al. describe a causal relationship of the presence of naïve CD8+ T cells and claimed that in acute and convalescent cases of Covid-19, peak disease severity was associated with a lack of naïve CD8+ T cells and suggested the relationship may be causal by virtue of their less proliferative and responsive capacity (12).

Diao et al. also noted elevated PD-1 and TIM-3 expression on CD8+ T cells in recovering patients and proposed the cells may be exhausted in the latter stages of the disease, with CD4+ cells showing elevated PD-1 expression in worsening disease (10). Zheng et al. noticed a kinetic where the total number of Natural Killer (NK) cells and CD8+ T cells would decrease with worsening disease, and upregulate the marker of exhaustion NKG2A, but they claim with successful treatment with lopinavir-ritonavir the counts would then increase and NKG2A expression would decrease during the convalescent period (13). Zheng et al. did not find lymphopenia in their cohort, however they noted that a non-exhausted subgroup of PD-1- CTLA4- TIGIT- CD8+ T cells were significantly reduced in percentage in patients with severe Covid-19 compared to mild and moderate (14). Westmeier et al. sought to characterize the proteins that cause cytotoxicity and possibly immunopathological organ damage, and they found in young (29–79) patients CD8+ T cells activated *ex vivo* following convalescence produced granzymes A, B, and perforin at greater levels than healthy donors despite expressing PD-1, suggesting the exhaustion was not functional despite PD-1 expression (15). However, the CD8+ T cells of the elderly group (ages 80–96) did not upregulate the cytotoxic markers Granzyme B and Perforin as the younger group did (ages 29–79), indicating age-related exhaustion (15). They conclude PD-1 expressing CD8+ cells should not be ‘misclassified’ as exhausted, however they state that PD-1 therapy may improve virus control (presumably in the elderly group) but also may “exaggerate the immunopathology in the lungs and other organs” (15). We agree with this therapy in regard to aged patients but also propose that cytotoxic function may be overexuberant in younger populations and that withholding differentiation may, to an extent, temper Granzyme and Perforin expression. Because of the possibility raised, testing T cell modulators in pre-clinical animal models is advised.

Ueland et al. recapitulate the findings by Diao et al. and report that sTIM-3, an exhaustion marker associated with chronic infections including HIV, Hepatitis B, Hepatitis C, and pulmonary tuberculosis, had a rise in expression in severe (ICU) patients, which correlated with the measured degree of pulmonary infiltration and the cardiac marker NTproBNP (16). Ueland et al. hypothesize that T cells are traveling to these organs and are responsible for their harm, but temporarily upregulate sTIM-3 as “a mechanism to prevent persistent and overshooting T-cell activation, which could harm the host” (16). They still, however, claim their findings suggest that T-cell activation and exhaustion play a role in Covid-19 and posit T-cell targeted treatment options may be of interest (16). Varchetta et al. report an increase in TIM-3 and CD69 expression in CD8+ T cells and note the extent of CD8+ T cell lymphopenia was significantly greater in patients that succumbed to Covid-19 (17). They describe this as a hyperactivated/exhausted state and observed that during recovery TIM-3 and CD69 expression on T cells fell and CD8+ lymphocyte count rose. They offer TIM-3, PD-1, and NKG2A as “druggable molecules” that may be used to “unleash antiviral activity” (17).

In an autopsy series, Nienhold et al. describe two “immunopathological reaction patterns” (18). The first is characterized by lung infiltration of CD8+ PD1+ T cells that they speculate are causing diffuse alveolar damage (DAD) but better viral control, and they question whether the PD-1 positivity denotes exhaustion, but no conclusions are drawn. The other immunopathological pattern is characterized by significantly higher CD4+ T cell lung infiltrates and a higher viral load (18). They conclude this suggests CD8+ T cells indeed contribute to viral clearance but may exert a degree of end organ damage (18). Schurink et al. found either one of two types of T cell infiltration in all autopsies: a CD8+ infiltrate causing DAD, or a CD4+ interstitial infiltrate with exudative diffuse alveolar damage and bronchopneumonia (19).

#### Too Many Licenses to Kill

Multiorgan tissue damage is present in severe cases of Covid-19 and Multisystem Inflammatory Syndrome in Children (MIS-C), possibly contributed to by T cells activated by a superantigenic-like insert (20). After activation, such T cells may be capable of migration to peripheral tissues following their differentiation and acquisition of cytotoxic ability conferred by effector differentiation (20). Cheng et al. argue that virtue of the superantigenic insert, infection would prime self-reactive T cells (20). Combined with the apparent downregulation of FOXP3 in CD4+ CD25+ T cells and their expression of FASL in Covid-19 patients (21), this could bode poorly for organs and immune-privileged sites. Following Covid-19 even canonically immune-privileged sites like the eye have inflammatory infiltrates (22) along with organs like the heart (19). Israelow et al. observe hyperactivation of CD8+ cells and their infiltration into the lung in a murine model of Covid-19 (23). Indeed, an increase in the frequency of effector cells has been shown in one analysis of T cell physiological correlates of Covid-19 disease severity; CD8+ T cells in severe cases expressed increased levels of Granzyme and Perforin than in patients with mild disease (16). Whether this represents increased effector function or a marker for exhaustion in Covid-19 is unknown considering that in the Covid-19 disease state, since Kalfaoglu et al. show even PD-1 was not inducing lymphocyte exhaustion (21). Taken with the recent findings discussed previously, we propose testing the alternative hypothesis put forward by Westmeier et al, that the expression of granzyme and perforin may engender greater end organ damage by virtue of their function (15). These insights and observations suggest exuberant activation in Covid-19 may facilitate the differentiation of T cells. Overexuberant acquisition of effector function may be targeted through CD95’s nonapoptotic signal *via* Akt (24).

#### Evidence of Absence (Hyperactivation and Perhaps Death)

Zinzula describes the crucial role of the type I interferon responses in Covid-19 in respect to the viral ORF8 protein (25). When this protein, which downregulates MHC I, is ablated with the frameshift mutation Δ382, patients experience mild illness (25). In a study of patients with the mutation, Young et al. find these patients exhibit higher levels of IFN-γ, TNF-α, IL-2, and IL-5, which they contextualize as associated with T cell activation (26). Illustrating the importance of an early response, Tan, et al. show early T cell responses were associated with rapid viral clearance and mild disease and suggested an early IFN‐ γ producing CD4+ T cell response recognizing ORF7/8 may be implicated in viral control (27). Without early control Sars-Cov-2 may accumulate and create pathogenesis through cytopathic and immune-mediated mechanisms. Habel et al. describe an irreconcilable amount of CD8+ “bystander activation … by some mechanism”. They also observe, in convalescence, a paucity of circulating effector subsets, and describe it as either due to 1) limited clonal expansion and differentiation or 2) T cell effectors are being driven to sites of virus-induced pathology and to apoptosis (28). In a transcriptome analysis Zhang et al. found that the CD8+ naïve T cell subset did not restore to the levels of healthy donors in convalescence, and that a subset of CD8+ effectors expressing GNLY had highly cytotoxic function, became high and remained high even in convalescence (29). Furthermore, using Gene Set Ontology, they found statistically significant, stepwise increases in the programs for 1) apoptosis and 2) migration in T cells from patients with Covid-19 (healthy donor/moderate/severe), which led them to conclude “T cells in severe patients likely underwent migration and apoptosis” (29).

Taken together with the proposed presence of a superantigenic-like insert by Cheng et al., we propose that attenuation of the honest expression of MHC I by ORF8 may allow superantigenic accumulation to a point to when the adaptive compartment is finally presented antigen, there is an atypically massive viral protein and superantigenic burden in the pipeline which may hyperstimulate, lead to AICD, overexuberant differentiation, tissue migration (as per the alternative hypothesis put forth by Habel, et al.), and cytotoxic function. Indeed, De Biasi et al. find production and release of cytokines they describe as similar to “a polyclonal, superantigen-driven T-cell activation” and observed increased levels of IFN-γ in the plasma of Covid-19 patients (30). They also noted the increased expression of CD57 on CD8+ T cells of Covid-19 patients, which they claim denotes a susceptibility to activation-induced cell death and a lack of proliferative capacity (30). However, Focosi et al. note there is preservation of cytokine secretion after stimulation of CD57+ lymphocytes (31). Furthermore, Chattopadhyay et al. have noted CD8+ T cells “expressing high levels of Perf were uniformly bright for CD57” (32).

With the findings by Bellesi et al, of increased CD95 expression, we believe there may be a contributing element of apoptosis to the lymphopenia observed in Covid-19, but this is a challenge to prove as cells which apoptose by this mechanism do not remain for functional read-outs. Exogenously withholding differentiation may **1)** reduce the expression of the cytotoxic proteins like granzyme and perforin that Westmeier, et al. theorize contribute to end organ damage; **2)** maintain CD62L expression and other functional markers associated with less differentiation which may keep T cells from peripheral migration; and **3)** prevent CD95-mediated death, which has been shown to predominantly occur in differentiated effector memory lymphocytes rather than naïve, stem cell memory, and central memory subsets of differentiation (24).

### Aberrant CD4+ T Cell Differentiation in Covid-19

Aberrant effector differentiation has been described in CD4+ T cells in severe cases of Covid-19 as well (21). Kalfaoglu et al. describe an aberrantly differentiated subset of CD4+ CD25+ FOXP3- T cells they call “hyperactivated T-cells” which become multifaceted Th1-Th2 effector cells rather than Tregs due to the repressed expression of FOXP3 (21). These CD4+ CD25+ T cells ‘vigorously’ proliferate, downregulate FOXP3, and express FasL in a dysfunctional differentiation pathway induced by Covid-19 (21). The authors also state the CD25+ ‘hyperactivated T-cells” also expressed PD-1, but noted it was not able to control or suppress their function and hypothesize CD80 may be suppressing PD-1 function (21). We would like to bring this insensitivity to exhaustion in context to the observations and assumptions of exhaustion in studies cited previously. Such insensitivity to PD-1 in these aberrant cells represents exuberant function, and the FasL expression may represent a liability. For example, FasL expression on CD8+ T cells is known to confer a paracrine T-cell fratricidal liability *in vivo* (33), and a paracrine T-cell to T-cell FasL-Fas signal was also shown to enhance differentiation of naïve CD8+ T cells by concurrently stimulated memory CD8+ T cells in an effect that was mitigated by a FasL blocking antibody (24). This paracrine effect of FasL also pushed naïve CD8+ T cells to express dramatically increased Granzyme B and Ifn-γ levels (24).

Much like their CD8+ counterparts, Non-apoptotic signaling of Fas differentiates murine CD4+ T cells as well (34); Cruz et al. show that LZ-FasL treatment during *in vitro* stimulation of CD4+ cells, enhances differentiation from naïve to CD44^hi^ CD62L^lo^ effector memory for wild type CD4+ T cells (34). Differentiation was induced in Fas palmitoylation-deficient (C194V) cells, (which are replete in non-apoptotic Fas signaling but have a palmitoylation defect which prevents the apoptotic signal of Fas and therefore the apoptosis) but not for CD4+ *lpr* cells which are completely deficient in Fas signaling (34). Additionally, non-apoptotic signaling of Fas has been shown to reduce the frequency of FOXP3+ CD4+ Tregs in mice (34). *lpr* mice completely deficient in Fas signaling were crossed to express a version of Fas that was replete in non-apoptotic signaling but that had the palmitoylation defect (C194V) had a decrease in Tregs from the higher frequency of that in *lpr* alone, to a normal/near wild-type frequency, which may suggest non-apoptotic Fas signaling *in vivo* may reduce the frequency of FOXP3 expressing CD4+ cells (34). Furthermore, Akt signaling in human CD4+ T cells represses FOXO1- a key transcription factor that enables FOXP3 expression and function (34–36). CD4+ T cells, like CD8s, have effector differentiation withheld during stimulation with an AKT inhibitor (37, 38) and also retain functional FOXO1 (38). It seems warranted to test whether FOXP3 expression can be maintained by inhibition of the Fas/AKT pathway in the Covid-19 disease state.

### CD95 Sensitivity Following Activation Leading to Differentiation

**Figure 1:**
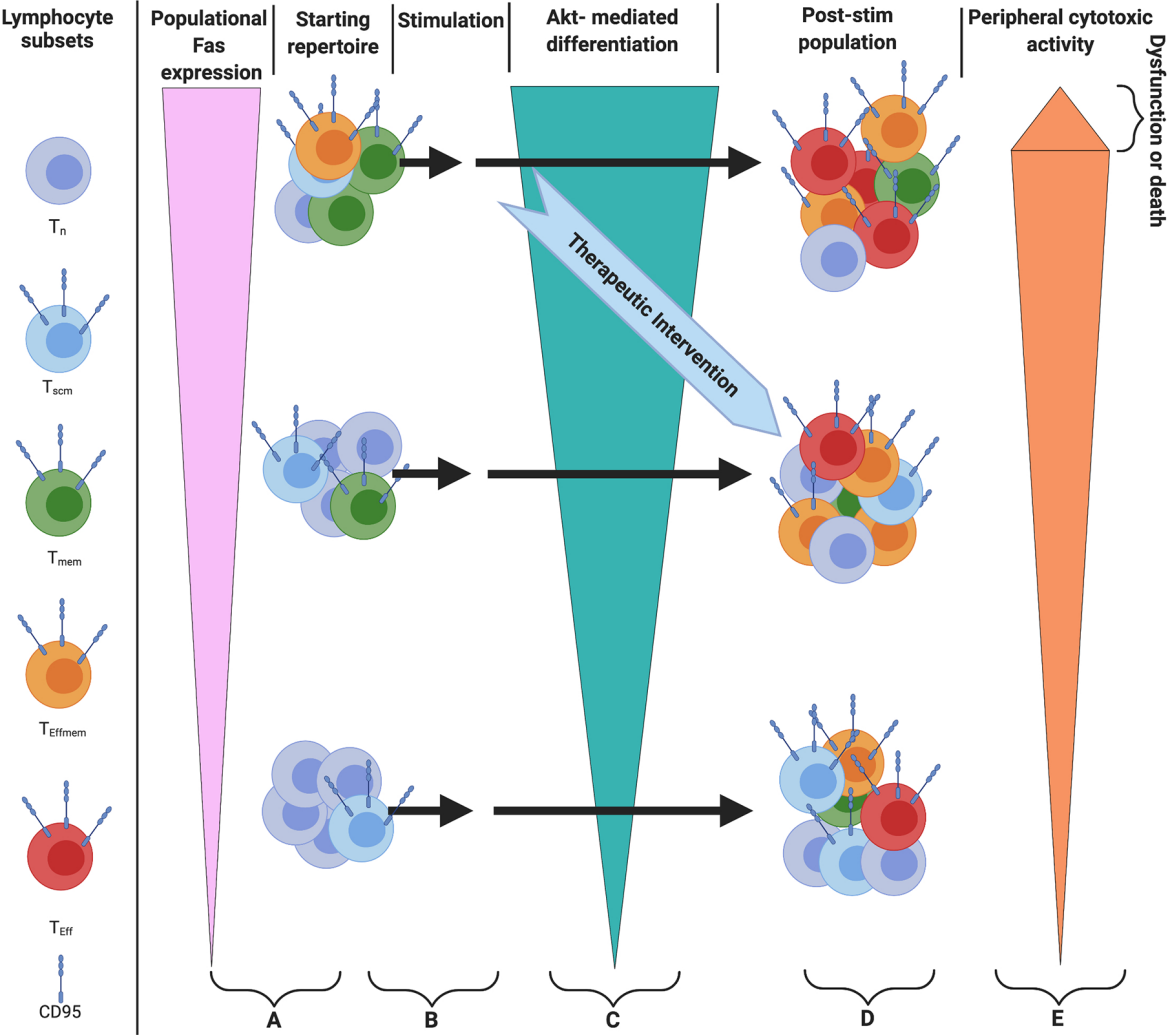
Model of *in vivo* therapy targeting differentiation with either AKTi or Fas/FasL blocking antibody **(A)** Starting populations of T cells and relative Fas expression. Note: patients with greater levels of differentiation would be more likely to benefit from this therapy by virtue of greater expression of Fas **(B)**. Superantigenic stimulation of T cells **(C)** Akt-mediated differentiation of T cells. Note: Stimulation can serially occur, and differentiation can be attenuated by providing an Akt inhibitor or Fas/FasL blocking antibody *in vivo* **(D)**, The population of cells after stimulation **(E)** cells with greater levels of differentiation will have higher levels of cytotoxic function, with the most terminally differentiated likely to show true exhaustion as referenced in the text.

In parallel with lymphopenia in severe cases of Covid-19, the machinery responsible for T cell death and differentiation are enriched as well. Bellesi et al. showed CD-95 was highly expressed on the T cells of Covid-19 patients (5), and Mathew et al. identify CD95 as a marker of aberrant CD8+ cells in an ‘immunotype’ associated with severe Covid-19 (39). Both the expression of CD95 on T cells and the sensitivity to CD95 stimulation are rapidly inducible through T cell activation (24), and Cheng et al. argue T cells are activated by a superantigenic motif on the Spike protein of SARS-Cov-2 that is highly similar to staphylococcal enterotoxin B (20). Activated T cells are especially sensitive to the CD95-mediated differentiation signal (24). Interestingly, a non-apoptotic CD95 signal was shown to cause differentiation and effector function in activated (CD3/CD28 stimulated) murine and human CD8+ T-cells in a dose-dependent manner with leucine zipper tagged Fas ligand (LZ-FasL), and this differentiation signal was carried through Akt (24). Interestingly, the death induced by trimerized CD95 Ligand (CD95L/CD178) was shown to be predominantly limited to the terminally differentiated cells rather than the Naïve and Central Memory (24). Furthermore, death and differentiation were withheld during *in vitro* stimulation and expansion of T cells by use of an AKT inhibitor even in the presence of LZ-FasL, revealing a dominant effect of AKT inhibition for the mitigation of CD95 signaling (24). Considering this mechanism of widespread activation, T cells not specific to SARS-COV-2 may differentiate, achieve expression of functional cytotoxic proteins, and die, possibly *via* a CD95 mediated component (**Figure 1**).

### Who Will be The First to Akt?

Akt inhibition as a potential therapeutic for Covid-19 has been proposed in the past. Fagone et al. describe a transcriptomic profile in which Akt signaling was associated with disease progression (40), and Somanath discusses a similar rationale of modulating immune-mediated inflammation: that Akt inhibition is justified on the basis of an anti-inflammatory effect exerted by CD4+ T cells that may differentiate into Effector T regulatory cells in the ARDS lung (41). While we concur, we would like to supplement this argument further with discussion of the effects on the differentiation and acquisition of effector function and the inclusion of nonapoptotic CD95 upstream signaling. Akt inhibition has also been proposed on the basis of an anti-viral effect (42–44). However, it appears that in presenting stages of Covid-19 disease antiviral agents seem to have questionable therapeutic benefit, possibly due to the disease entering an immunopathological stage of exuberant lymphocytic response (19). Sorbera et al. claim SARS-Cov-induced apoptosis *via* a caspase-dependent mechanism may suggest inhibition of the Fas/FasL interaction may have efficacy in Sars-Cov-2, but the rationale disregards differentiation (45). Finally, in a CRISPR-Cas9 lentiviral vector knock out of GM-CSF in CAR T cells, Fas expression was significantly inhibited, which the authors argue would reduce CAR-T apoptosis (46), and GM-CSF inhibition trials are undergoing. Considering 1) the role the immune system may have in Covid-19 pathogenesis, 2) the observation that FasL is upregulated (21), and 3) that Fas signaling induces Akt-mediated differentiation, investigation is warranted.

### Physiological Type I Interferons Required: An Aetiological Subset of Covid-19 Disease

While we propose the attenuation of an exuberant effector differentiation may dampen end organ damage, there is a caveat to the general application of this proposed mechanism that requires nuance in the diagnosis and management of patients with distinct aetiologies. Lee and Shin discuss the heterogeny of type I interferon responses and attribute the cause of such discrepancies as due to timing of the observations (early vs. late) and severity of disease (mild/moderate/severe) (47). They propose a feed-forward mechanism found by Lee et al. where Type I IFNs break a tolerance induced by TNF (48). Lee et al. posit that an early IFN-I response can control viral replication, but a delayed response can cause pathological inflammation (48). Indeed, an interferon deficit can permit high levels of detectable virus in the blood (49). Illustrating this heterogeneity, Lee and Shin cite a trial which found administered IFNα had a protective effect if given early and a detrimental effect if given late (47, 50). Furthermore, Bastard et al. describe individuals predisposed to severe Covid-19 with autoantibodies against type I interferons representing about 10% of a cohort of severe patients (51), and Zhang et al. identify inborn errors of Interferons (including TLR3, IFNAR1, and IFNAR2) in 3.5% of patients in a cohort with life-threatening Covid-19 (52). We believe studying the role of CD95 in a murine model of Covid-19 without such inborn or acquired errors may not recapitulate the autoantibody or inborn errors of IFN seen in a subset of patients but would rather be more comparable to the pathological experience in a patient without such inborn defects in Interferon or autoantibody responses. We cannot comment on the T cell kinetics or differentiation states of such patients, or whether altering T cell differentiation would be a boon for such patients as insufficient data exists, however Zhang et al. propose it would be helpful to identify such patients early or even before infection so they may have their deficiencies supplemented with Interferon when an efficacious therapy is found (52).

### Mitigating the T Cell Physiological Consequences of Covid-19

CD4+ and CD8+ T cells post Covid-19 were on the whole, less comprised of naïve and memory subsets in survivors vs. healthy donors, while the former had a lesser proportion of naïve cells on a background of lymphopenia (53). When taking this finding from Yang et al. of a diminished proportion of Naïve CD4+ and CD8+ T cells post- Covid-19 (53) along with the finding from Moderbacher, et al. that naïve CD8+ T cells are associated with lesser disease severity (12), we must consider the consequences of reinfection if the pool of naïve T cells significantly diminishes after infection and outcomes are influenced by the presence of naïve CD8+ T cells. In this scenario, it would be best to preserve lesser-differentiated populations of T cells by blocking CD95-mediated T-cell death and differentiation *in vivo*, which is precedented in a murine-human xenograft model of CAR-T adoptive immunotherapy; CAR-T cells transduced with a recombinant Fas receptor lacking an intracellular signaling domain were substantially more capable of inducing durable tumor responses and showed increased *in vivo* persistence, presumably by virtue of their attenuated differentiation and death (54). Also, blocking T cell effector acquisition *in vitro* during TCR stimulation and expansion with a CD95 blocking antibody or with an AKT1/AKT2 (AKT VIII) inhibitor, has been shown to allow tumor-specific CD8+ T cells to proliferate without differentiating, thereby greatly increasing their numbers and withholding premature apoptosis (24). Furthermore, on a cell-for-cell basis, T cells with their differentiation withheld by Akt inhibition were superior at eradicating established human and murine tumor after adoptive transfer in both a murine and murine-human xenograft models of adoptive T cell immunotherapy (24, 37, 38). Akt inhibition also maintained a higher percentage of CD62L+ CD44- CD8+ T cells (Tn or Tscm) after stimulation, and Fas signaling induced with LZ-FasL, a trimerized CD95 ligand, differentiated naïve T cells in a dose-dependent fashion (24). While we are aware that stimulation and expansion *in vivo* vs*. ex vivo* are entirely different contexts, we propose the differentiation and death signal and susceptibility of T cells to CD95 is similar. It would be advisable to further characterize the effect *in vivo*.

## Conclusion

The role of an Akt-mediated CD95 signal as a causal factor rather than a marker in this aberrancy warrants exploration since CD95 could be implicated in the exuberant effector differentiation and death of T cells in severe Covid-19 (21). Given the dual function of CD95, CD95-mediated differentiation and death may be advancing T cells to greater effector acquisition, fewer numbers, and immune dysregulation (**Table 1**). This may be a pathological state yielding tissue damage due to superantigenic stimulation of T cells not specific to SARS-Cov-2. Whether CD95-mediated death and/or differentiation is pathogenic can be tested in murine models of severe/lethal Covid-19, like the K18 hACE2 model (55). Preclinical animal models of severe Covid-19 that recapitulate a type I interferon competent and lymphopenic experience may be useful for the investigation of a strategy that seeks to preserve T cells and reduce their dysfunction by preventing their death and differentiation.

**Table 1:** Findings of Key References.

Finding	Reference
Lymphopenia in severe Covid-19	(1–4)
Increased T-cell Fas expression in Covid-19	(5)
Cytokine storm hypothesis under question	(7)
Evidence of a preferential CD8+ paucity in severe Covid-19	(10–12)
Markers of exhaustion/overactivation that are possibly harmful	(10, 13–17)
Pathology reports of harmful immune function	(18, 19)
T cells are stimulated broadly in Covid-19	(20, 30)
T cells are overactivated and aberrantly differentiated in Covid-19	(21)
In Covid-19 CD4+ T cells downregulate FOXP3	(21)
Stimulated immune cells are infiltrating organs	(19, 22, 23)
Evidence of apoptosis and cell migration	(29)
Akt signaling downregulates FOXO1	(34, 35)
FasL causes differentiation, effector function, and apoptosis	(24, 33, 34)
FasL signals through Akt	(24, 38)
FOXO1 helps retain FOXP3 expression	(34–36)
T cells treated with Akt inhibitor retain FOXO1	(38)
Akt and FasL Blockade prevent T-cell differentiation and apoptosis	(24, 37, 38, 54)

## Author's Note

This work is dedicated to Alvin E. Marcus.

## Author Contributions

AJL wrote the first draft and revision. AJL and RBP wrote the manuscript and weighed the evidence. All authors contributed to the article and approved the submitted version.

## Conflict of Interest

AJL has filed a patent application pertaining to the methods described here.

The remaining author declares that the research was conducted in the absence of any commercial or financial relationships that could be construed as a potential conflict of interest.
